# Antibacterial Activity of Polyphenols: Structure-Activity Relationship and Influence of Hyperglycemic Condition

**DOI:** 10.3390/molecules22111913

**Published:** 2017-11-06

**Authors:** Yixi Xie, Jing Chen, Aiping Xiao, Liangliang Liu

**Affiliations:** 1 Institute of Bast Fiber Crops, Chinese Academy of Agricultural Sciences, Changsha 410205, China; xieyixige@xtu.edu.cn (Y.X.); chenjingcaas@yahoo.com (J.C.); aipingxiao@yahoo.com (A.X.); 2 College of Chemistry, Xiangtan University, Xiangtan 411105, China

**Keywords:** antibacterial activity, diabetes, glycation, infection, polyphenol

## Abstract

Polyphenols are plant-derived natural products with well-documented health benefits to human beings, such as antibacterial activities. However, the antibacterial activities of polyphenols under hyperglycemic conditions have been rarely studied, which could be relevant to their antibacterial efficacy in disease conditions, such as in diabetic patients. Herein, the antibacterial activities of 38 polyphenols under mimicked hyperglycemic conditions were evaluated. The structure-antibacterial activity relationships of polyphenols were also tested and analyzed. The presence of glucose apparently promoted the growth of the bacterial strains tested in this study. The OD600 values of tested bacteria strains increased from 1.09-fold to 1.49-fold by adding 800 mg/dL glucose. The polyphenols showed structurally dependent antibacterial activities, which were significantly impaired under the hyperglycemic conditions. The results from this study indicated that high blood glucose might promote bacterial infection, and the hyperglycemic conditions resulting from diabetes were likely to suppress the antibacterial benefits of polyphenols.

## 1. Introduction

Polyphenols are secondary metabolites of plants, and are well known as natural antioxidants. Based on their chemical structures, polyphenols can be classified as flavonoids and non-flavonoids [1]. Flavonoids possess C_6_-C_3_-C_6_ carbon structures consisting of two phenyl rings (A and B) and a heterocyclic ring (C). According to the hydrogenation degree of the heterocyclic ring and the connection site of ring B, flavonoids can be further classified into several subclasses, such as flavones, flavonols, flavanones and isoflavonoids [2]. Non-flavonoids mainly include stilbenes, chalcones, anthraquinones, ellagitannins, ellagic acids and phenolic acids [3].

Polyphenols can be ingested by humans from the consumption of fruits, vegetables, and plant derived beverages. The consumption of diets rich in polyphenol have usually been associated with beneficial effects to human health [4]. Despite controversies, epidemiological studies suggest that dietary polyphenols could lower risk of cardiovascular disease, prevent obesity, cancer and type 2 diabetes, attenuate brain aging and Alzheimer’s disease, as well as maintain gut health [5,6]. These benefits have usually been associated with their diverse biological activities, such as anti-oxidation, anti-inflammation, anti-bacteria, enzyme inhibition, glycation inhibition, immunomodulation and miRNA interference [7,8,9]. Among these bioactivities, the antibacterial activities have attracted much interest due to the potential in dealing with the drug-resistant bacteria that are insensitive to conventional antibiotics [10]. Polypenols, especially flavonoids, have been suggested to exert their antibacterial effects in three ways; namely, direct killing of bacteria, synergistic activation of antibiotics, and attenuation of bacterial pathogenicity [11]. More importantly, flavonoids have been shown to be capable of inactivating efflux pump, destabilizing cytoplasmic membrane, and inhibiting β-lactamases and topoisomerase, and thus can prevent the development of antibiotic resistance in bacteria [12].

Diabetes mellitus is characterized by persistent high levels of glucose in the blood. This chronic hyperglycemia is apt to induce damage to various organs, especially the eyes, kidneys, nerves, heart, and blood vessels [13]. The development of diabetes is also associated with increased risks of cancer, cardiovascular disease, vascular dementia, Alzheimer’s disease, and some other metabolic diseases [14]. Additionally, the clinical data have shown that diabetic individuals are more susceptible to common infections than non-diabetics [15]. Compared to non-diabetic infections, the rehabilitation periods for diabetic infections are longer, and associated with higher mortality rate. In addition, a recent cohort study has shown that the risk of severe infection was increased among type 2 diabetic patients, and was not reduced by statin therapy [16]. Moreover, it has been found that the bacterial characteristics in the urinary tracts of diabetic patients with *Escherichia coli* infection were almost the same as those in non-diabetic ones [17].

In our previous study, we verified that the anti-oxidative effects of dietary polyphenols were diminished in the context of type 2 diabetes [18,19]. Herein, aimed at understanding the possible connection between the severity of infection and the impaired beneficial effects of dietary polyphenols in diabetes, the influence of hyperglycemic condition on the antibacterial activity of 38 polyphenols was investigated.

## 2. Results and Discussion

### 2.1. Characterization of the Glycation Products

In the process of glycation of proteins, fluorescent advanced glycation products (AGEs) such as vesperlysines A, B, C, pentosidine and pyrropyridine can be used as indicators of the glycation degree [20]. As shown in Table 1, the fluorescence intensities of gBSA and gBp at 460 nm were both significantly increased compared to the controls, indicating the formation of fluorescent glycation products after incubation. However, some glycation products of proteins were non-fluorescent, and could not be detected by fluorescent spectra. Hence the colorimetric determination of frucotosamine residues by using NBT was performed. As shown, the E_DMF_ value of BSA was only 1.84 mM, whereas that value for gBSA was increased by about 12 fold to 20.71 mM. The E_DMF_ value of gBP was also more than three fold of that of BSA. Moreover, since the glycation of proteins could modify the functional groups of chromophores, the effects of glycation on the UV spectra of BSA and BP were also investigated. Similar to the increase of fluorescence intensities, the absorbance at specific wavelengths was also increased after glycation, indicating the onset of AGE formation of proteins [21].

### 2.2. Antibacterial Susceptibilities of Polyphenols

A disc diffusion assay is usually applied to evaluate the antimicrobial susceptibility in vitro. Therefore, the potential antibacterial capacities of polyphenols were qualitatively screened using this method. The area of the inhibition zone for the tested polyphenol is positively correlated with its antibacterial effect. As shown in Table 2, 15 polyphenols show apparent inhibitory effects on the growth of certain bacteria (Φ > 8 mm), and the sensitivities of different bacteria to the same polyphenol are varied. Compared to the positive controls of antibiotics, most of the polyphenols showed weaker activities, except phenolic acids with antibacterial effects on VP comparable to antibiotics. However, unlike the broad-spectrum bactericidal effects of antibiotics, the antibacterial effects of polyphenols were selective. For example, hesperetin showed considerable antibacterial activity against the four Gram-negative bacteria (EC, ST, ES and VP), but no activity against the Gram-positive bacterium SA. Meanwhile, the remarkable inhibition zones of resveratrol were seen for EC, ST, SA and VP (9~12 mm), but not for ES. Similarly, selective antibacterial effects have been found by disc diffusion test for six antibacterial flavonoids from the aerial parts of *Pterocaulon alopecuroides*, which exhibited antibacterial activities only against the Gram-positive bacteria [22].

### 2.3. Minimum Inhibitory Concentration (MIC) Values and Structure-Activity Relationships

Since the disc diffusion tests only give qualitative results, and may not be suitable for comparative studies on the antibacterial activities of polyphenols, the quantitative MIC values of the antibacterial polyphenols were determined. The structure-activity relationships were discussed based on both the qualitative and quantitative results. As shown in Table 3, baicalein and myricetin show the most significant antibacterial effects among the tested flavonoids. Baicalein has a pyrogallol structure on ring A (5, 6, 7-OH) and myricetin also has a pyrogallol structure on ring B (3′, 4′, 5′-OH), which in combination indicated that the pyrogallol structure was an indictor for potent antibacterial activity for flavonoids. Additionally, all the flavonols and flavanones with antibacterial activities have two hydoxyl substituents on C-5 and C-7 of ring A in common, such as quercetin, rutin, narigenin, and hesperitin. These results are in agreement with the previous report that these structures are associated with the antibacterial ability of flavonoids [23]. In addition, flavanones are more active than the corresponding flavones. For example, narigenin showed antibacterial effects on all the tested bacteria whereas apigenin showed almost no effect. This result may indicate that the saturation of the C_2_=C_3_ double bond increased the antibacterial activity. Comparing the activities between flavonoid aglycones and glycosides, it could be seen that the glycosides showed lower activity than aglycones. Moreover, resveratrol showed remarkable antibacterial activity, which vanished after the hydroxyl group at position 3 was substituted by a glucosyl group to form Piced. All the phenolic acids showed significant antibacterial activities, especially the pyrogallic acid, and the substituents possessing longer carbon chains offered the gallic acid derivatives stronger antibacterial activities.

### 2.4. Effects of Glucose on the Bacterial Growth

**Figure 1:**
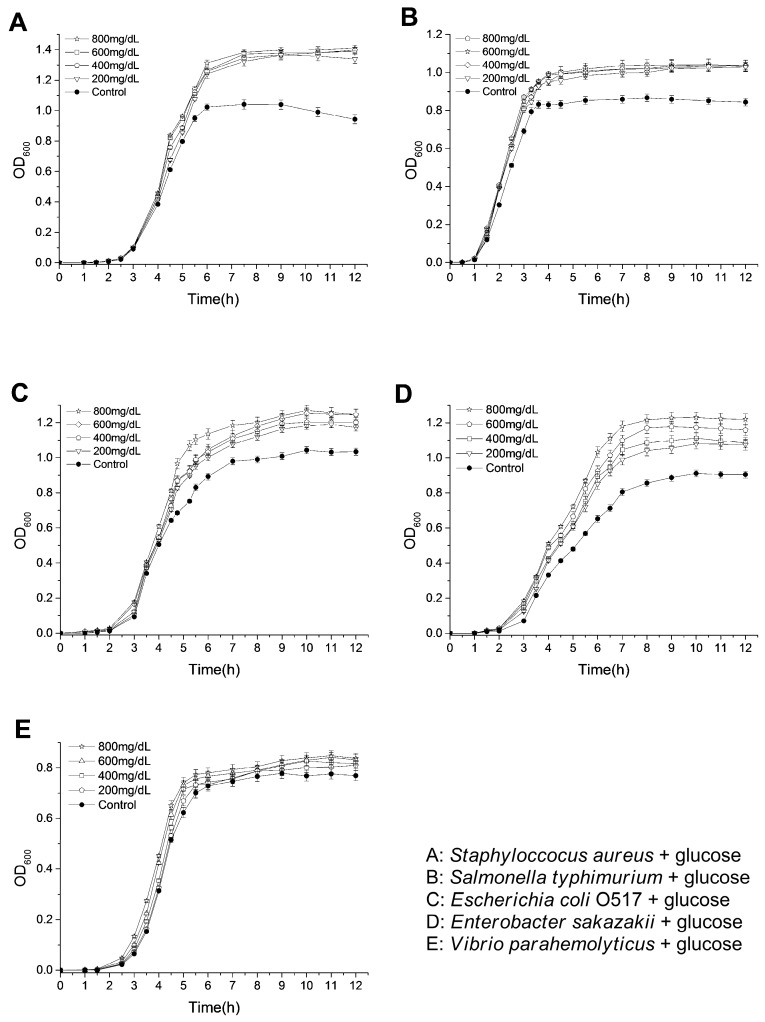
Effects of glucose on the growth of tested bacteria.

Diabetic patients are characterized with a higher blood glucose level than normal people. These glucoses are likely to be the nutrients for the growth of bacteria with infections. Herein, the tested bacteria were cultivated in Luria-Bertani (LB) media with glucoses at the range of 200 mg/dL to 800 mg/dL, and the OD_600_ values for the bacterial suspensions were monitored to evaluate the influence of glucose on the bacterial growth. As shown in Figure 1, glucoses promoted the growth of the bacteria in LB medium in varying degrees. For ST and SA, their OD_600_ values at stationary phases were significantly increased by only adding 200 mg/dL glucose (1.42-fold for SA and 1.22-fold for ST by adding 200 mg/dL glucose). When higher concentrations of glucoses were added, the increases of OD_600_ values were not apparent (1.49-fold for SA and 1.23-fold for ST by adding 800 mg/dL glucose). In addition, OD_600_ values at stationary phases of EC and ES were increased along with increasing glucose concentrations in LB medium (1.21-fold for EC and 1.35-fold for ES by adding 800 mg/dL glucose). However, the glucoses only slightly enhanced the growth of VP (1.09-fold by adding 800 mg/dL glucose). These results are consistent with the previous study, which showed that the addition of glucose (less than 1000 mg/dL) in both urine and Mueller-Hinton broth enhanced the growth rate and final bacterial yield for several *Escherichia coli* strains [24]. Recently, it was also found that the elevation of basolateral glucose concentration promoted growth of apical *S. aureus* in an in vitro airway epithelia-bacteria co-culture model [25]. The improved growth rate and cell density of *Lactobacillus* species have also been observed by increasing the glucose concentrations from 0.1% to 0.5% in a defined vaginal secretion-simulating medium with the presence of 0.005% MnCl_2_ [26]. Except for nourishing pathogenic bacteria, glucose might also play various important roles in infections. It might help the formation of a biofilm of bacteria, which is closely related to the occurrence of drug-resistance [27]. In addition, glycolysis is required for *S. typhimurium* infections of mice and macrophages, and the transport of glucose is required for the replications within macrophages [28]. Moreover, glucose sometimes even acts as an environmental signaling molecule, which can, for example, trigger ATP secretion from bacteria and regulate the transcription of invasion-associated genes of bacteria [29].

### 2.5. Antibacterial Activities of Polyphenols under Hyperglycemic Conditions

The simulated hyperglycemic conditions were created by adding glucose or glycated products into the LB medium, and the MIC of values of the most potent antibacterial polyphenols against the corresponding bacteria were subsequently determined. As shown in Table 4, with the presence of glucose, the MIC values of polyphenols were mostly increased compared to the control. For instance, the MIC values of bacalein for ES and SA were 0.5 mmol/L and 0.25 mmol/L, respectively, and both of them were doubled in the medium with glucose. In addition, for most of the tested polyphenols, the MIC values obtained in the medium by adding BSA, gBSA, BP, and gBP were larger than those in the control. These results indicated that the proteins, when added to LB medium, whether glycated or non-glycated, all inhibited the antibacterial effects of polyphenols. Moreover, the antibacterial effects of polyphenols in the LB medium with gBSA were generally weaker than those in the LB medium with BSA. Pyrogallic acid showed a MIC of 0.75 mmol/L for both ST and VP in BSA group, and this value was doubled in gBSA group. Comparing the MIC values of BP group with those of gBP group, it was also found that the gBP presence reduced the antibacterial activity of polyphenols more than BP did. Since glucose has been shown to promote the growth of bacteria, it is not surprising that the polyphenols in the medium with glucose showed inhibited antibacterial activities. Proteins, especially serum albumins, usually bind with polyphenols, and it has been suggested that only the non-protein-bound fraction of polyphenols is microbiologically active [30]. Hence, the presence of proteins reasonably posed a negative influence on the antibacterial activity of polyphenols. Other researchers have also found the impairment of antibacterial activity by plasma protein binding, which is attributed to the prevention of intra-bacterial uptake of antibiotics [31]. According to our previous study, the glycation of plasma proteins lowers the binding affinities for polyphenols, and the glycation of human serum albumins is believed to reduce the binding affinities for acidic drugs such as polyphenols and phenolic acids [19]. However, with the concentrations used in this study (>0.1 mmol), the ratios of polyphenol-BSA binding and polyphenol-gBSA binding are different. Thus, the gBSA itself, being rich in highly reactive AGEs, may play important roles in diminishing the antibacterial effects of polyphenols. It has been revealed that AGEs are also produced, metabolized, and accumulated even in short-lived bacteria cells, which are usually secreted by the energy-dependent efflux pump systems [32]. Hence, the AGEs in gBSA are likely to interact with the possible AGEs receptors in bacteria and stimulate the efflux pump systems which are responsible for the removal of polyphenols from the bacteria cells [33]. Accordingly, the significantly decreased antibacterial effects of polyphenols in gBP can also be attributed to AGEs. Moreover, the highly reactive carbonyl species produced during the glycation process are able to trap polyphenols and inhibit their antibacterial activities [34].

### 2.6. Effects of Polyphenols on the Growth of Bacteria under Hyperglycemic Conditions

**Figure 2:**
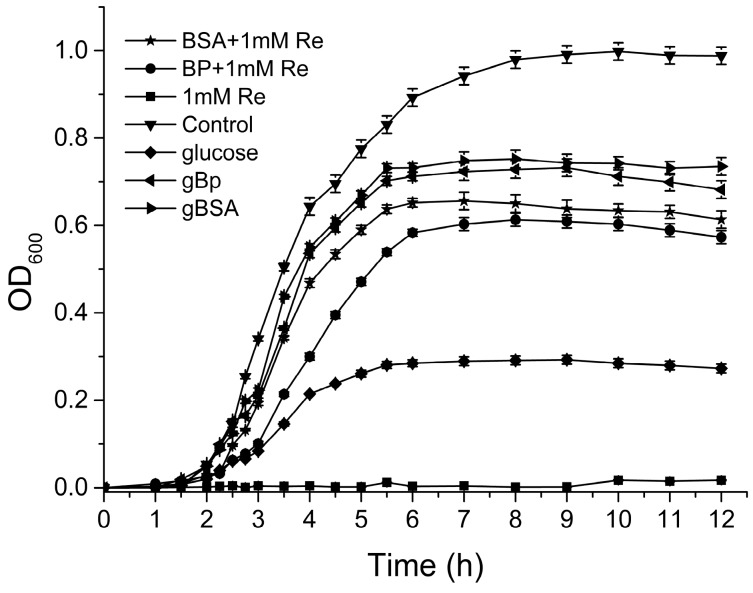
Effects of resveratrol (Re) on the growth curve of EC in different environments.

Two potent antibacterial polyphenols, resveratrol (Re) and pyrogallic acid (PA) were further investigated on their inhibitory effects against the growth of EC and SA, respectively. As shown in Figure 2, with the addition of 1 mM resveratrol (MIC) the growth of EC was completely inhibited in the pure culture medium. However, the glucose apparently increased the survivability of EC in response to Re. In addition, the supplements of proteins and plasmas also significantly weakened the inhibitory effects of Re on the growth of EC. The growth rate and the final cell density of EC in BSA group were lower than those in gBSA group, indicating that gBSA impaired the antibacterial effect of Re. It has been reported that the growth rate of enterobactin-producing *E. coli* was significantly increased in an iron-limited medium with addition of glycated BSA, which was attributed to the enhanced iron availability by protein glycation [35]. Comparing the growth in BP group with that in gBP group, the antibacterial performance of Re was reduced in the hyperglycemic environments.

**Figure 3:**
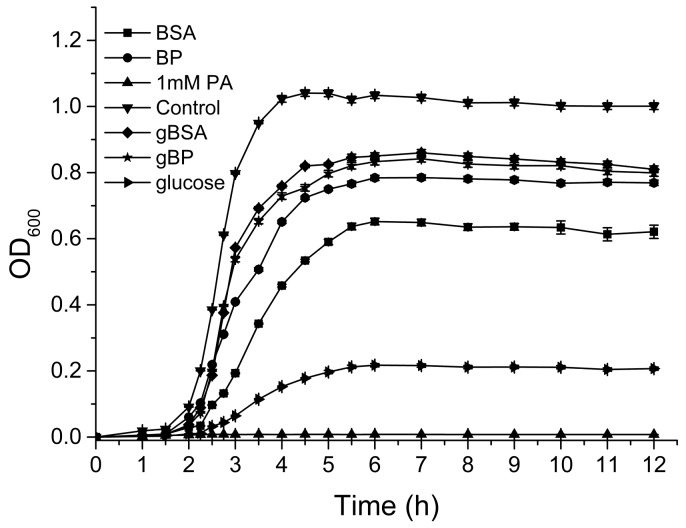
Effects of pyrogallic acid (PA) on the growth curve of SA in different environments.

The effects of PA on the growth of SA in different culture mediums are shown in Figure 3. PA at a concentration of 1 mM completely restrained the growth of SA in the pure medium. The glucose and proteins negatively influenced the antibacterial effects of PA. Additionally, the negative influences of gBSA and gBP were stronger than those of BSA and gBP, respectively, which also supported the conclusion that the antibacterial performance of polyphenols was suppressed under hyperglycemic conditions. These results indicated that high blood glucose may create a hotbed of bacterial infection, and the hyperglycemic conditions results from diabetes are likely to suppress the antibacterial benefits of polyphenols.

## 3. Materials and Methods

### 3.1. Chemicals and Reagents

Flavone, 6-hydroxy flavone, 3-hydroxy flavone, 7-hydroxy flavone, 6-methoxy-flavone, 6-methoxy flavanone, naringenin and naringin were purchased from Tokyo Chemical Industry Co., Ltd. (Tokyo, Japan). Biochanin A, genistein, apigenin, puerarin, wogonin, catechin (C), epicatechin (EC), luteolin, daidzein, daidzin, resveratrol, piceid and neohesperidin-dihydrochalcone were purchased from Aladin Co. Ltd. (Shanghai, China). Galangin, kaempferol, quercetin, myricetin, baicalein and chrysin were purchased from Wako Pure Chemical Industries (Osaka, Japan). All other polyphenols were obtained commercially from Shanghai Tauto Biotech CO., Ltd. (Shanghai, China). Peptone, yeast extract, agar, 1-deoxy-1-morpholino fructose (DMF) and bovine serum albumin (BSA) were purchased from Sigma-Aldrich (St. Louis, MO, USA). Nitrotetrazolium Blue chloride (NBT) was obtained from Sinopharm Chemical Reagent Co., Ltd. (Beijing, China). Kanamycin and carbenicillin were purchased from Shanghai Xinya Pharmaceutical Co., Ltd. (Shanghai, China). Fetal bovine serum (BP) of no phage and low endotoxin was from Sijiqing Biological Engineering Materials Co., Ltd. (Hangzhou, China). All other reagents and solvents were analytical grade and used without further purification.

### 3.2. Apparatus

UV Absorbance was read on a Nano Drop 2000 spectrophotometer of Thermo Fisher Scientific (Wilmington, DE, USA). The fluorescence spectra were recorded on a HITACHI F-7000 spectrophotometer in a 1 cm quartz cell (Tokyo, Japan). The pH measurements were performed on a PHSJ-3F pH-meter of Shanghai Precision & Scientific Instrument Co., Ltd. (Shanghai, China). The sterile experiment operations were carried out on a super-purifier clean bench of Suzhou purification equipment Co., Ltd. (Suzhou, China).

### 3.3. Glycations of BSA and BP

Glycation of BSA was carried out according to a rapid thermal glycation method [36]. Briefly, 50 mg/mL BSA was incubated with 0.5 M glucose in 0.2 M PBS (pH 7.4) for 7 days at 50 °C. Glycated BSA was collected and purified by ultrafiltration, then redissolved in isometric PBS solution. BSA incubated without glucose was used as the control group. Glycation of Bp was carried out by incubating BP sterilized by filtration (0.22 μm) with 8 mg/mL glucose (sterilized under 120 °C for 20 min) for 21 days at 37 °C. BP incubated alone for 21 days at 37 °C was used as the control group.

### 3.4. Determination of the Glycation Products

The glycation products were quantified by measuring the contents of fructosamine residues, determined by the method in the literature, with slight modification [37]. DMF at concentrations between 0 and 1 mM containing 50 mg/mL BSA was used for calibration. Contents of fructosamine residues in samples were monitored by comparison to the standard curve (*R*^2^ > 0.99) and expressed by DMF equivalent concentrations (E_DMF_, mM). The UV spectra of the samples were scanned from 250 nm to 800 nm, and fixed wavelength data at 330, 360 and 400 nm were obtained. The fluorescence spectra of the glycated BSA (gBSA) and glycated BP (gBP) were measured in the wavelength range of 350–700 nm upon excitation at 355 nm and the fluorescence intensities (FI) at 460 nm were recorded. Samples were diluted for measurements if necessary. The measures were performed in triplicate, and the results were found to be reproducible within experimental error.

### 3.5. Antibacterial Assay

The antibacterial activities of polyphenols against five common pathogenic bacterial strains were tested: one Gram-positive bacteria: *Staphyloccocus aureus* ATCC 12600 (SA); four Gram-negative strains: *Vibrio parahemolyticus* ATCC 17802 (VP), *Escherichia coli* O517:H7 ATCC 43895 (EC), *Salmonella typhimurium* ATCC 14028 (ST), *Enterobacter sakazakii* ATCC 51329 (ES). All the isolates were provided by Dr. Yu Zhao of Shanghai Normal University. The paper disc diffusion method was employed to determine the antibacterial activities of polyphenols. In brief, a 100 μL suspension of tested microorganism (10^9^ CFU/mL) was evenly spread on Luria-Bertani (LB) agar plates. Then, sterilized filter paper discs (Φ = 6 mm) soaked in 0.1 mol tested polyphenols (dissolved in DMSO) were placed on the inoculated plates. After incubation at 37 °C for 24 h, the diameters of the inhibition zones were measured by rulers. Kanamycin and carbenicillin were used individually as positive controls, and DMSO without polyphenols was used as a negative control. Each assay was repeated at least twice. The polyphenols showing apparent inhibition zones in disc diffusion assay were employed to investigate the antibacterial activity under hyperglycemic conditions by determination of minimum inhibitory concentration (MIC) values. The MIC was defined as the lowest concentration of polyphenol at which no bacterial growth was observed after incubation. The MIC values of polyphenols were determined using microdilution tests with LB broth, and were evaluated in the range of 0.1 mmol/L~2.5 mmol/L. In a typical assay, bacteria reaching their expotential phase were added into sterilized LB broth to obtain the culture broth with bacterial concentration of 1.0 × 10^5^ CFU/mL. 80 μL Bp (or BSA, gBSA, gBp) was separately added to 680 μL culture broth in 2 mL tubes; then, 40 μL polyphenol with different concentrations was added into each tube, and fully mixed. LB broths without bacteria were used as negative controls, and culture broths without polyphenols were used as positive controls. All the samples were placed in a 37 °C incubator for 24 h. After that, the MICs were determined by the microplate method [38]. To examine the effects of polyphenols on the bacterial growth rate and behavior under hyperglycemic conditions, the growth curves of the five bacteria in the presence of glucose were firstly studied. LB broth with glucose in the range of 200 mg/dL~80 mg/dL were prepared in 2 mL culture tubes, and 1.0 × 10^5^ CFU/mL of tested bacteria were inoculated. The bacteria were allowed to grow in a shaking incubator (200 rpm) at 37 °C. The Optical Density at 600 nm (OD_600_) values of the bacterial suspensions were then recorded by spectrophotometer at predetermined intervals. The inoculated LB broth without glucose was used as a control. Secondly, the effects of selected polyphenols on the growth curves of polyphenol-sensitive bacteria in different simulated hyperglycemic environments were investigated. Briefly, 80 μL glucose (800 mg/dL), gBSA or gBP was added into 680 μL inoculated LB broth, 40 μL polyphenol solution was then added to achieve a concentration of 1 mmol/L. Bacteria incubation and OD_600_ measurement were the same as in the above method. The bacterial growth curves in the presence of polyphenol along and together with BSA or BP were also investigated. Inoculated LB broths without polyphenols were used as controls. The measuring of samples was performed in triplicate.

## 4. Conclusions

In the present study, we have demonstrated that glucose increased the growth rate of five common pathogenic bacteria to different degrees in vitro. The antibacterial performances of polyphenols were found to be significantly impaired in the hyperglycemic conditions simulated to the diabetic state. These results supported the hypothesis that high blood glucose creates a hotbed of bacterial infection, and the hyperglycemic conditions resulted from diabetes are likely to suppress the antibacterial benefits of polyphenols. This hypothesis may provide another angle for interpreting why there is an enhanced risk of infection for diabetic patients, and why diabetic infections are always difficult to treat. Additionally, it is also helpful for effectively utilizing dietary polyphenols in maintaining the health of diabetic patients. Nevertheless, further in vivo studies are still needed to validate the present conclusions.

## Figures and Tables

**Table 1:** Detection results of the glycation products.

Samples	Fluorescence Intensities (FI 460 nm)	UV Absorbance			E_DMF_ (mM)
		330 nm	360 nm	400 nm	
BSA	22 *	0.054	0.023	0.006	1.84
gBSA	390	0.129	0.071	0.021	20.71
BP	12	0.024	0.012	0.003	1.04
gBP	95	0.079	0.034	0.011	3.23

* Relative Standard Deviations (RSD) of the results were less than 0.03.

**Table 2:** Results of antimicrobial susceptibility tests for polyphenols.

Subclass	(1 μM)/Bacteria	Subtitutions			Inhibition Zone (Φ, mm)				
		OH	OCH_3_	Others	EC	SA	ST	ES	VP
*Flavones*	Flavone				9 *	8	8	8	9
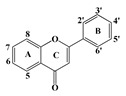	6-hydroxyflavone	6			-	-	-	-	-
	6-mehoxyflavone		6		-	-	-	-	-
	7-hydroxyflavone	7			-	-	-	-	-
	3-hydroxyflavone	3			-	-	-	-	-
	Bacalein	5, 6, 7			8	10	-	-	9
	Bacalin	5, 6		7-β-d-glucuronide	-	-	-	-	10
	Chrysin	5, 7			-	-	-	-	-
	Apigenin	5, 7, 4′			-	-	-	-	-
	Luteolin	5, 7, 3′, 4′			-	-	-	-	-
*Flavanones*	Flavanone				-	-	-	-	-
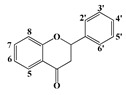	6-hydroxyflavanone	6			-	-	-	-	-
	4′-hydroxyflavanone	4′			-	-	-	-	-
	6-methoxyflavanone		6		-	-	-	-	-
	Narigenin	5, 7, 4′			8	11	8	9.5	11.5
	Narigin	5, 4′		7-neohesperidose	-	-	-	-	-
	Hesperitin	5, 7, 3′	4′		8.5	-	8	8.5	10
	Hesperidin	5, 3′	4′	7-rutinose	-	-	-	-	-
	Taxifolin	3, 5 ,7 ,3′ ,4′ ,5′			6.5	6.5	7.5	-	9.5
	Neohesperidin-dihydrochalcone	5, 7, 4′			-	-	-	-	-
*Flavonols*	Quercetin	3, 5, 7, 3′, 4′			8	9	8	8	-
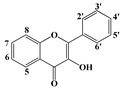	Myricetin	3, 5, 7, 3′, 4′, 5′			8	10	8	8	12
	Quercetrin	5, 7, 3′, 4′		3-o-β-d-Glucoside	-	-	-	-	-
	Fisetin	3, 7, 3′, 4′			-	-	-	-	-
	Rutin	5, 7, 3′, 4′			-	-	-	-	8
*Isoflavones*	Genistein	5, 7, 4′			-	-	-	-	7
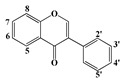	Formononetin	7	4′		-	-	-	-	-
	Biochanin A	5, 7	4′		-	-	-	-	7
	Puerarin	7, 4′			-	-	-	-	-
	Dadazein	7, 4′			-	-	-	-	-
	Dadazin			7-glucoside	-	-	-	-	-
*Stilbenes*	Piceid	5, 4′		3-glucoside	-	-	-	-	-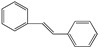									
	Resveratrol	3, 5, 4′			11	9	9	-	12
*Phenolic acids*	Gallic acid			R = H	7	-	8	-	-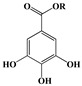	Propyl gallate			R = Propyl	10	7	-	10	19
	Methyl gallate			R = Methyl	9	7	-	8	12
	Ethyl gallate			R = Ethyl	9	-	-	8	20
	Pyrogallic acid				14	12	8	8	22
*Antibiotics*	Kana (0.5 μM)				22	20	25	22	20
	Carbenicilin (0.5 μM)				18	30	30	18	15

* Relative Standard Deviations (RSD) of the results were less than 0.03.

**Table 3:** Minimum inhibition concentrations of polyphenols on the tested bacteria.

Polypehols/MIC (mmol/L)	*E. coli* O517	*S. aureus*	ST	ES	VP
Flavone	2 *	2	2	2	1
Bacalein	0.5	1	1.5	1	0.5
Bacalin	2.5	>2.5	2.5	>2.5	>2.5
Quercetin	2	2	2	2	1
Myricetin	0.5	1	2	2.5	0.5
Taxin	>2.5	>2.5	>2.5	>2.5	2
Rutin	>2.5	>2.5	>2.5	>2.5	2.5
Narigenin	2.5	2.5	2.5	2.5	1
Hesperitin	1	>2.5	>2.5	>2.5	2
Resveratrol	1	2.5	2.5	2.5	1
Gallic acid	>2.5	>2.5	>2.5	>2.5	2.5
Propyl gallate	2	>2.5	>2.5	2.5	1.5
Methyl gallate	2.5	>2.5	>2.5	>2.5	2.5
Ethyl gallate	2	>2.5	>2.5	2.5	1.5
Pyrogallic acid	1	1.5	1.5	1.5	0.5
Kana	0.1	0.25	0.25	0.1	0.05
Carbenicilin	0.1	0.25	0.25	0.1	0.05

* Relative Standard Deviations (RSD) of the results were less than 0.03.

**Table 4:** Antibacterial activities of polyphenols in different environments.

Bacteria	Polyphenol	Control	MIC (mmol/L)				
			Glucose	BSA	gBSA	BP	gBP
*E. coli O517*	Myricetin	1 *	1	1.5	1.5	1.5	2.5
	Bacalein	0.5	0.5	0.5	0.75	0.75	1.5
	Pyrogallic acid	0.75	0.75	0.75	1	1	2
	Resveratrol	1	1.5	1.5	1.5	1.5	1.5
*E. sakazakii*	Pyrogallic acid	1.5	2	1.5	2	2	>2.5
	Bacalein	0.5	1	1	1	1	2.5
*S. typhimurium*	Pyrogallic acid	0.75	1	0.75	1.5	1	2.5
	Myricetin	1	1	1	1.5	1.5	2
*S. aureus*	Pyrogallic acid	1	1.5	1.5	2	1.5	>2.5
	Bacalein	0.25	0.5	0.5	0.75	0.5	2
*V. parahemolyticus*	Pyrogallic acid	0.75	1	0.75	1.5	1	>2.5
	Naringenin	0.75	0.75	1	1	1	>2.5

* Relative Standard Deviations (RSD) of the results were less than 0.03.
